# Identification and differential expression of serotransferrin and apolipoprotein A-I in the plasma of HIV-1 patients treated with first-line antiretroviral therapy

**DOI:** 10.1186/s12879-020-05610-6

**Published:** 2020-11-27

**Authors:** Sushanta Kumar Barik, Keshar Kunja Mohanty, Ashok Kumar Mohanty, Preeti Rawat, G. Gopal, Deepa Bisht, Shripad A. Patil, Rananjay Singh, Devesh Sharma, Srikanth Prasad Tripathy, Rekha Tandon, Tej Pal Singh, Srikanta Jena

**Affiliations:** 1 National JALMA Institute for Leprosy and Other Mycobacterial Diseases, ICMR, Tajganj, Agra, Uttar-Pradesh 282004 India; 2 National Dairy Research Institute, ICAR, Karnal, 132001 India; 3 Cancer Institute, Chennai, 600020 India; 4 National Institute for Research in Tuberculosis, ICMR, Chennai, 600031 India; 5 Sarojini Naidu Medical College, Agra, 282002 India; 6 Ravenshaw University, Cuttack, Odisha 753003 India

**Keywords:** HIV, AIDS, First line ART, Drug resistant, Drug respondent

## Abstract

**Background:**

Plasma proteins are known to interfere the drug metabolism during therapy. As limited information is available regarding the role of plasma proteins in HIV drug resistance during ART in HIV/AIDS patients, the present study aimed to identify and characterize the differentially expressed plasma proteins in the drug resistant and drug respondent groups of HIV-1 infected patients with > 6 years of first line ART.

**Methods:**

Four-drug resistant (treatment failure) and four-drug respondent (treatment responder) patients were selected for plasma proteomic analysis based on viral load and drug resistance associated mutations from a cohort study designed on the first line ART patients who were enrolled in the antiretroviral therapy center, Sarojini Naidu Medical College, Agra, India from December 2009 to November 2016.

After depleting high abundant proteins, plasma proteins were resolved using two-dimensional gel electrophoresis on IPG strips, pH range of 3–10. Spots were selected in the gel based on the density of staining which was common in the drug resistant and drug respondent groups separately. The fold change of each spot was calculated using image-J. Each protein spot was identified using the matrix assisted laser desorption/ionization-time of flight/time of flight (MALDI-TOF/TOF) after tryptic digestion. Peptide peaks were identified through flex analysis version 3.3, and a search against a protein data base using the internal Mascot. Gene ontology study was completed through STRING v.11 and Panther15.0.

**Results:**

Out of eight spots from 2D gel samples analyzed by MALDITOF/TOF, two proteins were found to have significant score (> 56) after Flex analysis. These two proteins were identified to be apolipoprotein A1 and serotransferrin. The fold change expression of these two proteins were analyzed in drug resistant and drug respondent group. Apolipoprotein-A1 and serotransferrin were observed to be expressed 1.76 and 1.13-fold more respectively in drug respondent group compared to drug resistant group. The gene ontology analysis revealed the involvement of these two proteins in various important physiological processes.

**Conclusion:**

Apolipoprotein A-I and serotransferrin were found to be expressed more in drug respondent group compared to drug resistant group.

**Supplementary Information:**

The online version contains supplementary material available at 10.1186/s12879-020-05610-6.

## Background

Human immunodeficiency virus (HIV) causes acquired immune deficiency Syndrome (AIDS) [1]. Innate and adaptive factors in human plasma regulate the activation of the HIV-1 genome after infection [2]. HIV drug resistance is a major problem and a threat in epidemic control [3]. There is no specific option to cure AIDS [4]. Clinically significant interactions between the drug and plasma proteins were observed. The pharmacological activity of anti-retroviral (ARV) drugs is dependent on unbound drug entering cells that harbor the human immunodeficiency virus. There has been concern that changes in protein binding could impact on antiviral activity and management. The study of pharmacological activity of antiretroviral drugs in HIV patients is very important. Changes in protein binding could impact antiviral activity in the host [5]. Plasma protein binding to the drugs and its effective modulation in drug metabolism help in drug discovery and development of new drug therapy [6]. The matrix-assisted laser desorption /ionization time-of-flight/time-of-flight (MALDI-TOF/TOF) MS analyses of fractioned human serum or plasma samples were carried out earlier [7]. Human saliva or serum proteins were identified through MALDI-TOF/MS for biomarker discovery [8, 9]. Plasma proteins are the main components of the human plasma proteome. It is very crucial to identify and delineate the proteins in each individual. In a diseased population, the gene or protein diversity study aid in discovery of clinical biomarker. The protein diversity related to the disease can be identified and evaluated easily in a population [10]. Therefore, we attempted to identify the plasma proteins in drug resistant (treatment failure) and drug respondent (treatment responder) groups of HIV-1 patients who were treated with first-line ART over 6 years.

## Methods

### Study subjects

The study included eight HIV-1 infected AIDS patients from a cohort of first line ART patients who were enrolled in the antiretroviral therapy center, Sarojini Naidu Medical College, Agra, India from December 2009 to November 2016. The initial criteria for inclusion was based on the immunological marker i.e. CD4^+^ count (< 350) and later based on the viral load and genotyping test for drug resistance associated mutations. These subjects were with 4 males and 4 females age ranging from 28 to 45 years old. The informed consents were obtained from all patients. A patient information leaflet was used for data collection [11]. First line ART was given as per the treatment guidelines directed by NACO, Govt. of India (NACO 2007, 2013). Briefly, the doses of zidovudine (250 to 300 mg twice daily), lamivudine (300 mg), tenofovir (300 mg), abacavir (300 mg), stavudine (30 mg), nevirapine (200 mg), efavirenz (600 mg) were given to each patient in different combination. The different regimen combination of drug respondent and drug resistant patients are given in the supplementary file 1.

### Isolation of plasma samples

Five ml of blood was collected aseptically by antecubital venipuncture in EDTA vacutainers. After incubating the blood samples at room temperature for 30 min to 1 h, samples were centrifuged at 2000 g at 4 °C. Supernatant was collected as plasma and stored at-80 °C for viral load estimation, genotyping and proteomic analysis.

### Viral load estimation

Viral load was estimated using the Abbott automated m2000rt instrument stationed at National Institute for Research in Tuberculosis, Chennai, India.

### Genotyping

The genotyping was carried out by the WHO dried blood spot protocol 2010 (WHO, 2010) [12]. The details of polymerase chain reaction (PCR) and primer conditions are given in the supplementary file-2.

### Viral load and mutations in HIV drug resistant group

The nucleoside reverse transcriptase inhibitors (NRTIs) and non-nucleoside reverse transcriptase inhibitors (NNRTIs) drug resistance mutations of the four patients were analyzed using the HIV drug resistance database, Stanford University, USA. The details of viral load, NRTIs and NNRTIs associated mutations analysis are given in the Table 1.

**Table 1:** Viral load, NRTI and NNRTI mutations

Sl No.	Viral load (RNA copies/ml)	NRTI mutation	NNRTI mutation
1	30,694 copies/ml	M41L, L74I, M184V,	V108I, Y181C, G190A, H221Y
2	103158copies/ml	None	None
3	6266 copies/ml	M184V, T215F	K103N, M230L
4	2950copies/ml	K65R, Y115F, M184V	K103S, V106M

### Reagents

Reagents used for high abundant protein depletion, 2D Gel electrophoresis and SDS-PAGE are given in supplementary file-3.

**Fig. 1:**
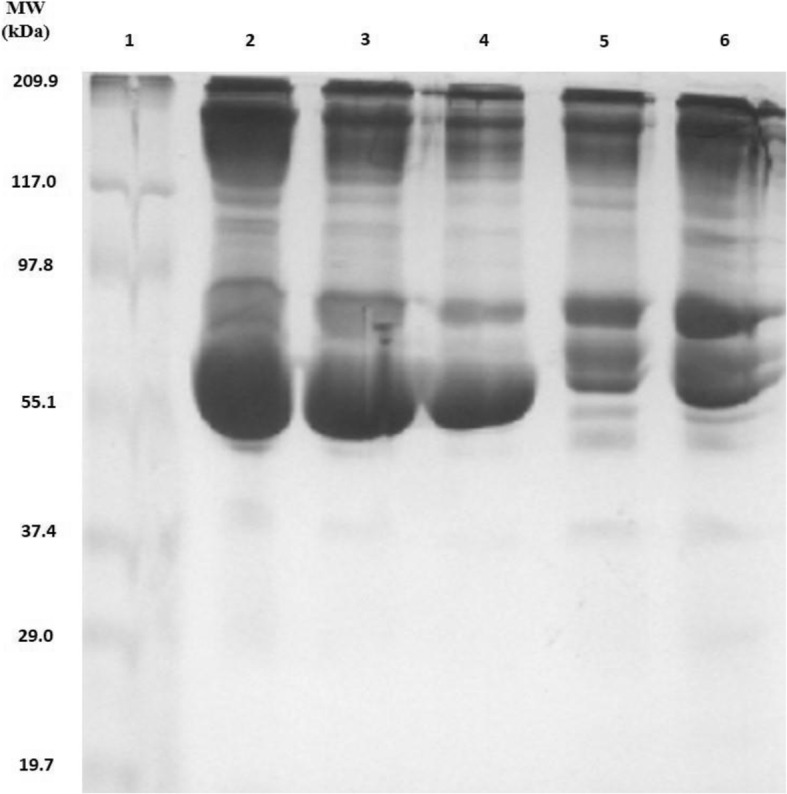
10% SDS-PAGE of plasma proteins extracted from HIV-1 infected human and their purification by Aurum serum mini kit (Bio-Rad, USA). Lane 1: Proteins marker; Lane 2, 3, 4: Before purification; Lane 5, 6: After purification.

Sixty μl plasma sample was taken for depletion of highly abundant proteins like immunoglobulins and albumins (Bio-Rad mini kit, USA). The 10% SDS-PAGE was performed with high abundant protein depleted samples. The representative picture is given in Fig. 1.

### 2-dimensional gel electrophoresis and tryptic digestion

Briefly, a 120 μg of protein sample was prepared in sample rehydration buffer. Passive rehydration of the protein was conducted for 22 h., followed by active rehydration in 8 steps such as 1. 50 V - 2 h-slow, 2.250 V − 0.15 h-slow, 3.1000 V-0.30 h-slow, 4.1500 V-0.30 h- slow, 5.2500 V-0.30 h-rapid, 6.4000-3 h-rapid 7.4000-4 h-rapid, 8.500-5 h-rapid. First dimensional gel electrophoresis was performed on a horizontal IEF apparatus (Bio-Rad, USA) and second dimensional gel electrophoresis was performed using 10% SDS-PAGE. The SDS-PAGE was stained in 0.2% Coomassie brilliant blue R-250. Tryptic digestion was performed by following a published protocol [13]. A Zip tip with C18 material was used for desalting of peptides. The desalted peptides were vacuum-dried.

### MALDI-TOF-TOF/MS analysis

Vacuum dried peptides were suspended in 10 μl of a 50% acetonitrile 0.1% formic acid solution. One microliter of samples and one microliter matrix consisting of a saturated solution of a-cyano-4-hydroxycinnamic acid prepared in 50% acetonitrile/0.1% formic acid were spotted on to the MALDI sample target plate of the Brucker Daltonics workstation. 1000shots and 5000 shots were taken for each sample using MALDI-TOF-TOF /MS (Bruker Daltonics, USA) and peptide peaks were analyzed using flex analysis version3.3. Proteins were identified in the Mascot [14]. Protein score greater than 56 with *p*-value< 0.05 are considered as significant score. Details of the raw data of the human apolipoprotein-A1 and serotransferrin are given in the supplementary file-4 and 5.

### Statistical analysis

The average density of individual spot detected after 2D-gel electrophoresis of 4 drug respondent and 4 drug resistant samples were measured. Then the ratio of the average density of individual spot of the drug respondent to the drug resistant sample was calculated. The ratio was represented as fold change expression. The mean fold change expression of identified proteins of both the groups were compared using student’s t-test. *P*-value less than 0.05 was considered as significant. Proteins were identified in the Mascot for MALDI-TOF-TOF/MS experiment. Protein score greater than 56 with *p*-value< 0.05 are considered as significant score.

## Results

In this study, the patients were classified in to two groups based on the drug resistance/viral.

copy numbers as drug resistant (having a viral load ≥1000 copies/ml) and drug respondent (having viral load (≤62 RNA copies/mL to target not detected levels of ≤40 RNA copies/ml). Viral genomes isolated from drug resistant patients had NRTIs and NNRTIs associated mutations in the reverse transcriptase gene of HIV-1. The drug resistant group considered as treatment failure and drug respondent group had no NRTIs and NNRTIs associated mutations in the reverse transcriptase gene of HIV-1. The drug respondent group considered as treatment success or virological success.

### 2-dimensional gel electrophoresis

**Fig. 2:**
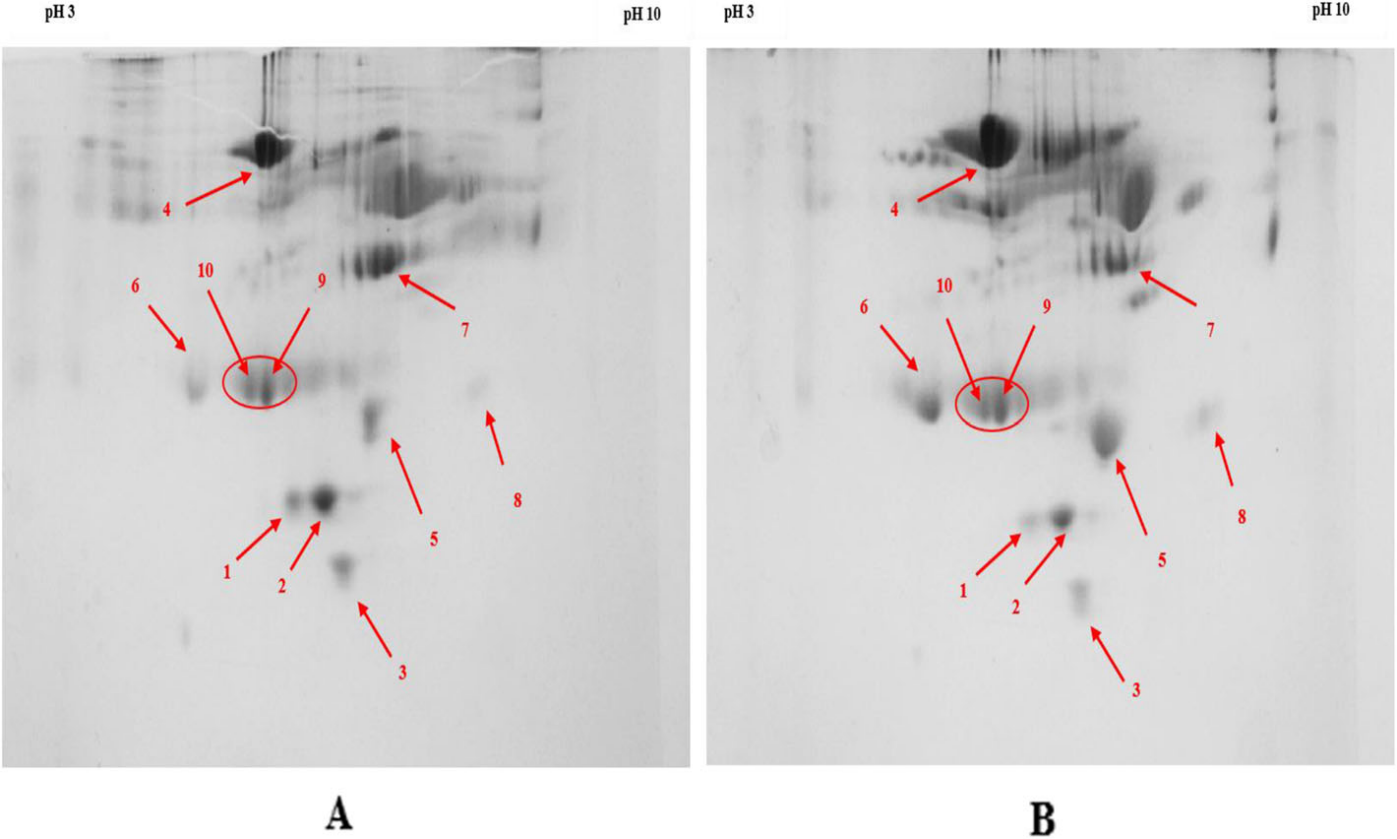
2D gels electrophoresis of purified proteins from human plasma samples of HIV-1 patient. **a** Treatment responder (control), (**b**) Treatment failure (test), Spots indicated by arrow were excised and analysed by MS. (Enriched spots 9 & 10 are taken as internal control).

**Fig. 3:**
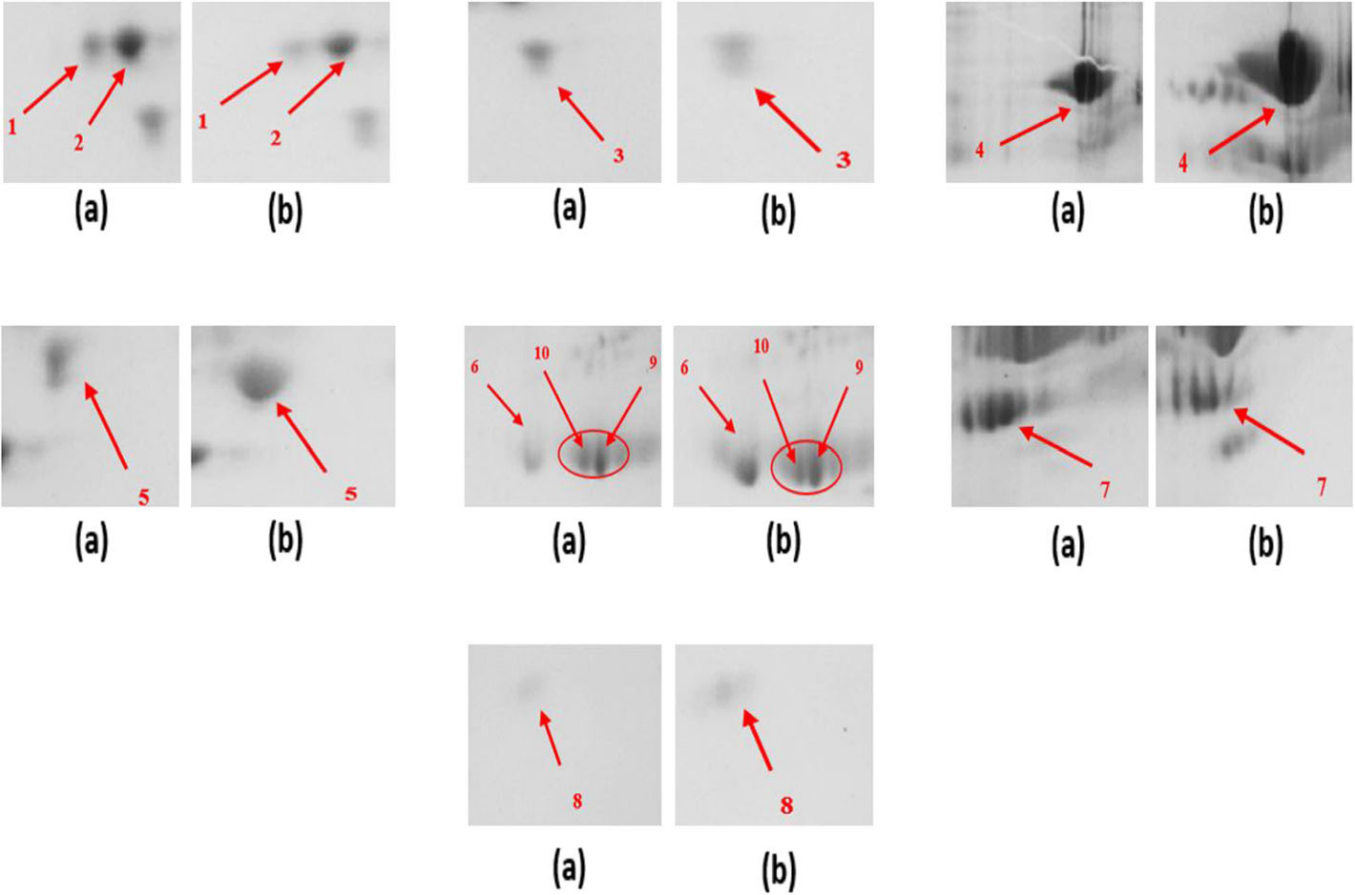
Magnified images of differentially expressed proteins spot: Magnified regions of 2D gels showing expression of protein (**a**) Treatment responder (control), (**b**). Treatment failure (test).

The representative pictures of 2-D gel electrophoresis are given in the Fig. 2. The magnified images of each spots are given in the Fig. 3. Eight protein spots were identified in the gel based on the density of staining which was common in the drug resistant and drug respondent groups separately. The fold change of each spot was calculated using image-J [15]. The average density of individual spot of 4 drug respondent and 4 drug resistant samples was measured. Then the ratio of the average density of individual spot of the drug respondent to the drug resistant samples was calculated. The ratio was represented as fold change expression.

### Identification of differentially expressed proteins by MALDI-TOF-TOF

**Fig. 4:**
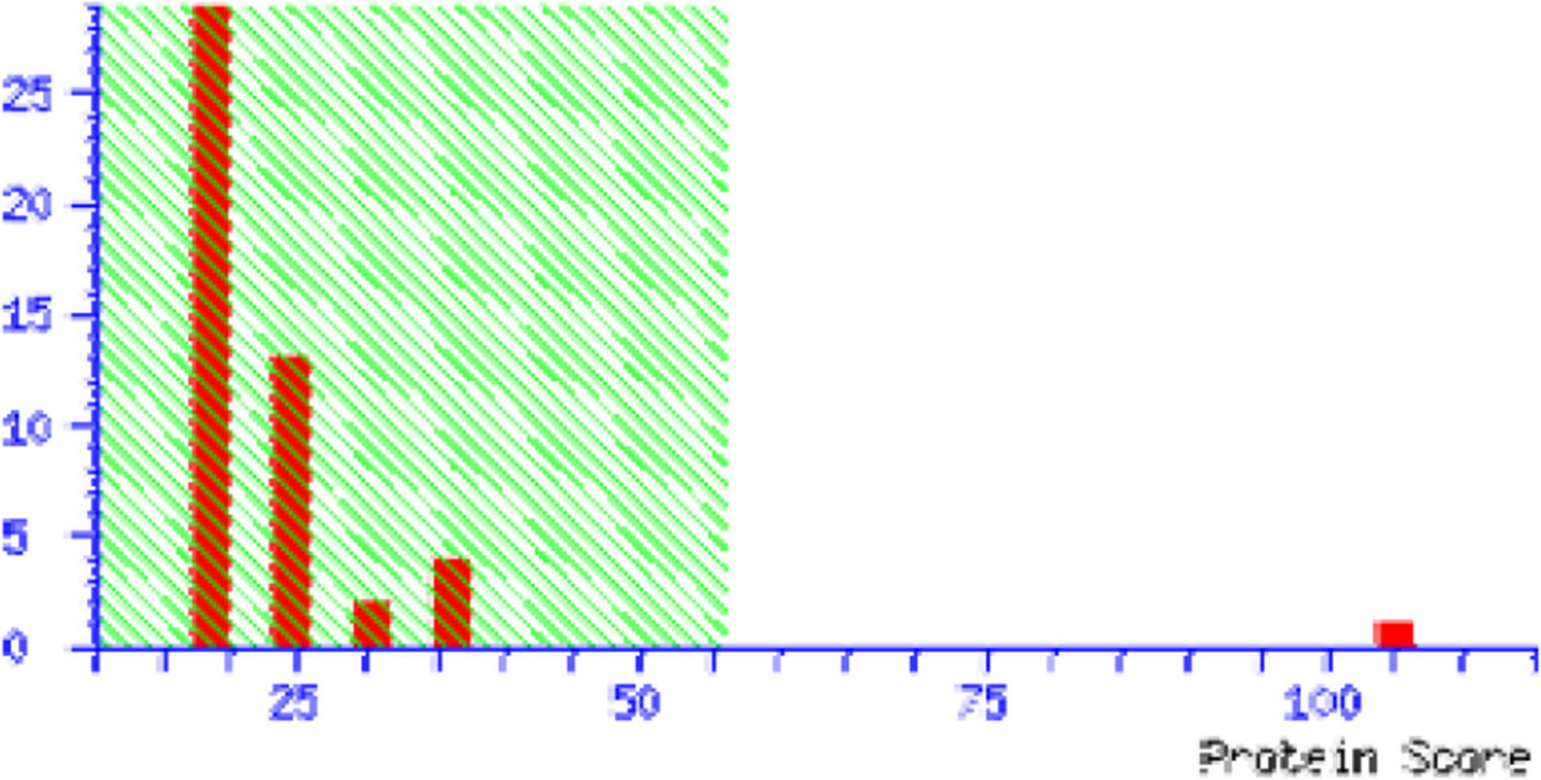
Mascot score histogram for serotransferrin. The protein score is − 10*Log(P), where P is the probability that the observed match is a random event. Protein scores greater than 56 were significant (*p* < 0.05). Score 105 for human serotransferrin.

**Fig. 5:**
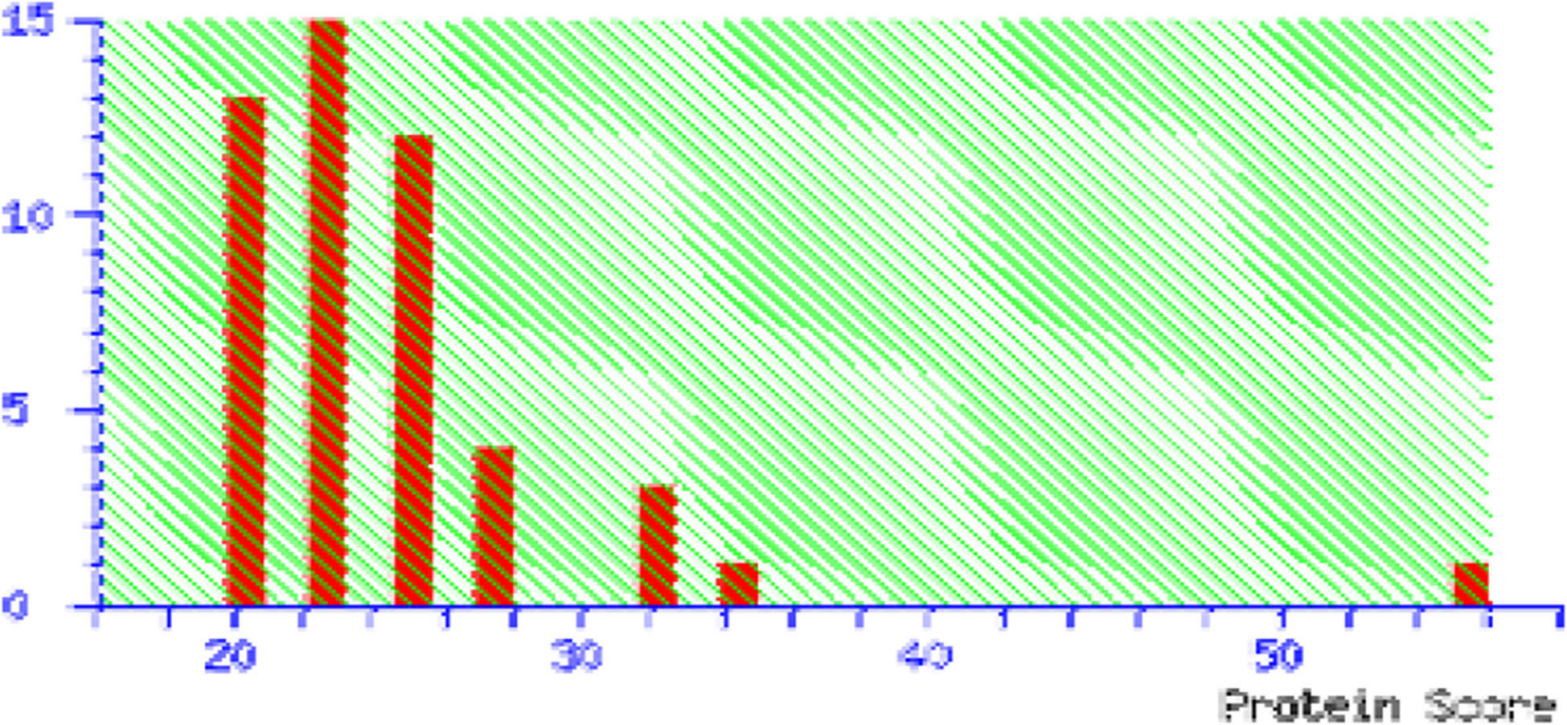
Mascot score histogram of apolipoprotein A-I. The protein score is − 10*Log(P), where P is the probability that the observed match is a random event. Protein scores greater than 56 were significant (*p* < 0.05). Score 56 for human apolipoprotein A-I (APOA1)

Eight common protein spots of drug respondent and drug resistant groups were taken for identification by MALDI-TOF/TOF. These spots were identified to be neuropeptide -B, colied-coil domain containing protein 104, keratin type-II cytoskeletal-I, serotransferrin, Apolipoprotein-AI, WD repeat containing protein, Ig kappa chain -V-III region, pH interacting protein. However, two proteins serotransferrin and apolipoprotein A-I were having significant (score > 56) through the Mascot. The serotransferrin of the respondent group was 1.13 fold change expression in comparison to the resistant group. The apolipoprotein A-I of drug respondent group was 1.76-fold change expression compared to the resistant group. The Mascot score histograms are given in Fig. 4 and Fig. 5 for serotransferrin and apolipoprotein-A1 respectively.

### Gene ontology study

The gene ontology of apolipoprotein-AI and serotransferrin was identified using STRING V.11 [16]. The biological process, cellular components and reactome pathways were analyzed by string11.0. The molecular function of the apolipoprotein-A1 was analyzed by panther-gene analyst 15.0 [17]. A protein network link was found in between apolipoprotein-A1 and serotransferrin. A detailed description of gene ontology analysis is given in the supplementary file-6.

## Discussion

Human plasma proteins have proven to be essential components of the blood and the study of these proteins are helpful to find out the function of the proteins involved in metabolism. The study of these proteins will be helpful for the development of diagnostic, prognostic or therapeutic markers in a disease. The pathogenicity of HIV is a global health concern. The study of protein-protein interactions, and protein drug interaction in HIV patients is crucial during the therapy. Some of the important proteins were earlier characterized in HIV patients. The APOA1 levels were measured in HIV-1 infected patients who are on nevirapine therapy. It was found that women had higher APOA1 levels than men. Apolipoprotein AI increased the level of sulfotransferase family2B member1gene activity and the Apolipoprotein AI synthesis was increased by Nevirapine. Apo-A1 different expression levels by aspartate aminotransferase and alanine aminotransferase were associated with sex dimorphic mechanism leading to nevirapine induced hepatotoxicity in HIV-1 patients [18]. Nevirapine increased the level of APO-AI in the HIV-1 infected patients [19]. Apo-AI was associated with apolipoprotein -AI binding protein to inhibit HIV replication [20]. In this study, lower level expression of APO-AI protein was observed in the drug resistant group as compared with the drug respondent group. Lower level expression of APO-AI might be the cause of high viremia and lower CD4 count in the drug resistant group of patients. Serotransferrins are iron-binding proteins that have antimicrobial properties [21]. Higher iron in serum was associated with HIV replication and increased the HIV infection [22]. Iron status was not an indication of disease severity in HIV-infected pregnant women in Malawi [23], but another finding was just reverse on iron overload was the severity in adults with HIV-1infected Caucasian population [24]. In our study findings, the level of serotransferrin was higher in the drug respondent group than in the drug resistant group. Further studies will be required to address the iron uptake in both groups.

## Conclusion

The two proteins named apolipoprotein A-I and serotransferrin were found to be differentially expressed in drug resistant and drug respondent group. Although, the fold change difference was not statistically significant between these two groups, Apolipoprotein-A-I and serotransferrin were noted to be correlated with virologic status and immune parameters such as CD4 counts. As the sample number was a limitation of this study, further investigation is required in this area to explore the protein biomarkers in drug resistant group.

### Gene Bank accession number

All submitted gene bank accession numbers are MG788713, MG788728, MG788738, MG 788748.

## Supplementary Information

**Additional file 1.** Different regimen and durations of drug respondent and drug resistant patients.

**Additional file 2.** Primers and polymerase chain reaction (PCR) conditions (Table M1, Table M2 and Table M3).

**Additional file 3.** IPG strips (7 cm, pH 3–10) (Bio Rad, USA), Sample rehydration buffer (BioRad, USA), Acetonitrile (Sigma, USA), LCMS grade water, Ammonium bicarbonate (BioRad, USA), Dithiothreitol (Sigma, USA), Iodoacetamide (Sigma, USA), Formic acid (Sigma, USA), Acetonitrile (Sigma, USA), Trypsin (Promega), Zip tip with C18 material (Millipore, Germany).

**Additional file 4.** Apolipoprotein A-I.

**Additional file 5.** Serotransferrin.

**Additional file 6.** Protein networks and Gene ontology of serotransferrin and apolipoprotein-A1 by STRING -V 11.0.

**Additional file 7.** Gene Bank accession numbers.

**Additional file 8:** **Figure S1.** Original image of 10% SDS-PAGE of plasma proteins extracted from HIV-1 infected human and their purification by Aurum serum mini kit (Bio-Rad, USA). Lane 1: Proteins marker; Lane 2, 3, 4: Before purification; Lane 5, 6: After purification. Fig. 2 (a) and (b): Original 2D gel electrophoresis of purified proteins from human plasma samples of HIV-1.

## Data Availability

All data generated by MALDI-TOF-TOF/MS and analysed by Mascot during this study are included in this manuscript and its supplementary information files. In addition, the partial pol gene sequences were released in the National Centre for Biotechnology Information (https://www.ncbi.nlm.nih.gov/nucleotide). Also, the partial pol gene sequences are given in the supplementary file-7. The original 1-D and 2D gel electrophoresis figures are given in the supplementary file-8.

## Abbreviations

HIV: Human immuno-deficiency virus
AIDS: Acquired immuno-deficiency syndrome
MALDI-TOF-TOF: Matrix assisted laser desorption/ionization -time of flight/time of flight
IPG: Immobilized pH gradient
IEF: Iso electro focusing
SDS-PAGE: Sodium dodecyl sulfate -polyacrylamide gel electrophoresis
NRTIs: Nucleoside reverse transcriptase inhibitors
NNRTIs: Non-nucleoside reverse transcriptase inhibitors
Apo-A1: Apolipoprotein-A1
