# Supplementary material for: Identification and differential expression of serotransferrin and apolipoprotein A-I in the plasma of HIV-1 patients treated with first-line antiretroviral therapy
Source: BMC Infect Dis. 2020 Nov 27;20:898. doi: 10.1186/s12879-020-05610-6 (PMC7694411; doi:10.1186/s12879-020-05610-6)
Supplement: Supplementary file 1 — Additional file 1. Different regimen and durations of drug respondent and drug resistant patients. [file 12879_2020_5610_MOESM1_ESM.docx]

| **Sl No.** | **Types of patients** | **Duration of regimen** |
| --- | --- | --- |
| **Drug respondent** | |  |
| 1 | TLE, TLN | 32 months 15days |
| 2 | SLE,ZLN, TLE,TLN | 53 months 27 days |
| 3 | SLE,ZLE,TLE | 65 months 2 days |
| 4 | ZLN,ZLE,TLN,TLE | 49 months 24 days |
| **Drug resistant** | | |
| 1 | TLE | 24 months 1 day |
| 2 | ZLN,ZLE | 75 months 11 days |
| 3 | ZLE, TLE | 41 months 5 days |
| 4 | SLN, TLN,TLE,ZLN | 61 months 13 days |

Supplementary file -1

Different regimen and durations of drug respondent and drug resistant patients
