# Supplementary material for: Identification and differential expression of serotransferrin and apolipoprotein A-I in the plasma of HIV-1 patients treated with first-line antiretroviral therapy
Source: BMC Infect Dis. 2020 Nov 27;20:898. doi: 10.1186/s12879-020-05610-6 (PMC7694411; doi:10.1186/s12879-020-05610-6)
Supplement: Supplementary file 2 — Additional file 2. Primers and polymerase chain reaction (PCR) conditions (Table M1, Table M2 and Table M3). [file 12879_2020_5610_MOESM2_ESM.docx]

**Supplementary file-2: Primers and polymerase chain reaction (PCR) conditions (Table M1, Table M2 and Table M3)**

**Table M1**

**HIV *pol* amplification and sequencing primers for DBS amplification (from PHAC WHO specialized lab, Ottawa, Canada):**

| Genomic region | Primer name | Sequence (5’ to 3’) |
| --- | --- | --- |
| Protease RT-PCR | PR1. | TGAARGAITGYACTGARAGRCAGGCTAAT |
| Protease RT-PCR | PR2 | Rev AYCTIATYCCTGGTGTYTCATTRTT |
| Reverse Transcriptase RT-PCR | RT1 | TTTYAGRGARCTYAATAARAGAACTCA |
| Reverse Transcriptase RT-PCR | RT2 | Rev CCTCITTYTTGCATAYTTYCCTGTT |
| Protease Nested PCR | PR3 | YTCAGRCAGRCCRGARCCAACAGC |
| Protease Nested PCR | PR4 | CTGGTGTYTCATTRTTKRTACTAGGT |
| Reverse Transcriptase Nested PCR | RT3 | TTYTGGGARGTYCARYTAGGRATACC |
| Reverse Transcriptase Nested PCR | RT4 | Rev GGYTCTTGRTAAATTTGRTATGTCCA |

**Table M2**

**Nested PCR: Cycling conditions for 1^st^ round PCR: Temp, Time, Steps/Cycles**

| Temperature | Time | Step/cycles |
| --- | --- | --- |
| 95 ^0^C | 15 min | Denaturation(1 cycle) |
| 94 ^0^C | 30 sec |  |
| 52 ^0^C | 30 sec | 35 cycles |
| 72 ^0^C | 2.5min |  |
| 72 ^0^C | 10 min | Final extension (1 cycle) |
| 4 ^0^ C | Hold |  |

**Table M3**

**Cycling conditions for 2nd round PCR: Temp, Time, Steps/Cycles**

| Temperature | Time | Steps/Cycle |
| --- | --- | --- |
| 95 ^0^C | 10 min | Denaturation ( 1 cycle) |
| 94 ^0^ C | 20 sec |  |
| 52 ^0^C | 30 sec | 35 cycles |
| 72 ^0^C | 2.5 min |  |
| 72 ^0^ C | 10 min | Final extension (1 cycle) |
| 4 ^0^ C | Hold |  |
