# Supplementary material for: Identification and differential expression of serotransferrin and apolipoprotein A-I in the plasma of HIV-1 patients treated with first-line antiretroviral therapy
Source: BMC Infect Dis. 2020 Nov 27;20:898. doi: 10.1186/s12879-020-05610-6 (PMC7694411; doi:10.1186/s12879-020-05610-6)
Supplement: Supplementary file 3 — Additional file 3. IPG strips (7 cm, pH 3–10) (Bio Rad, USA), Sample rehydration buffer (BioRad, USA), Acetonitrile (Sigma, USA), LCMS grade water, Ammonium bicarbonate (BioRad, USA), Dithiothreitol (Sigma, USA), Iodoacetamide (Sigma, USA), Formic acid (Sigma, USA), Acetonitrile (Sigma, USA), Trypsin (Promega), Zip tip with C18 material (Millipore, Germany). [file 12879_2020_5610_MOESM3_ESM.docx]

Supplementary file-3

Reagents

IPG strips (7cm, pH 3-10) (Bio Rad, USA), Sample rehydration buffer (BioRad, USA), Acetonitrile (Sigma, USA), LCMS grade water, Ammonium bicarbonate ( BioRad, USA), Dithiothreitol (Sigma, USA), Iodoacetamide (Sigma, USA), Formic acid (Sigma, USA), Acetonitrile (Sigma, USA), Trypsin (Promega), Zip tip with C18 material (Millipore, Germany).
