# Supplementary material for: Identification and differential expression of serotransferrin and apolipoprotein A-I in the plasma of HIV-1 patients treated with first-line antiretroviral therapy
Source: BMC Infect Dis. 2020 Nov 27;20:898. doi: 10.1186/s12879-020-05610-6 (PMC7694411; doi:10.1186/s12879-020-05610-6)
Supplement: Supplementary file 4 — Additional file 4. Apolipoprotein A-I. [file 12879_2020_5610_MOESM4_ESM.docx]

Supplementary file-4: Apolipoprotein A-I


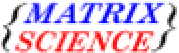
 **Mascot Search Results**

**User : sheev**

**Email : nallapeta@bdal.in**

**Search title :**

**Database : SwissProt 57.15 (515203 sequences; 181334896 residues)**

**Taxonomy : Homo sapiens (human) (20266 sequences)**

**Timestamp : 18 Feb 2020 at 08:50:23 GMT**

**Top Score : 56 for APOA1_HUMAN, Apolipoprotein A-I OS=Homo sapiens GN=APOA1 PE=1 SV=1** **Mascot Score Histogram**

Protein score is -10*Log(P), where P is the probability that the observed match is a random event. Protein scores greater than 56 are significant (p<0.05).


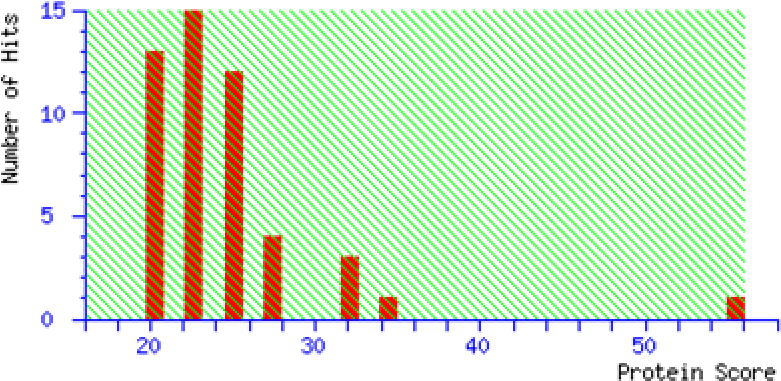


# Concise Protein Summary Report

Format As

6

Concise Protein Summary

Help

Significance threshold p<

0.05

Max. number of hits

100

Re-Search All

Search Unmatched

1. APOA1_HUMAN **Mass:** 30759 **Score:** 56 **Expect:** 0.057 **Matches:** 11 Apolipoprotein A-I OS=Homo sapiens GN=APOA1 PE=1 SV=1
2. FB5L3_HUMAN **Mass:** 11520 **Score:** 34 **Expect:** 7.7 **Matches:** 5

Putative fatty acid-binding protein 5-like protein 3 OS=Homo sapiens GN=FABP5L3 PE=1 SV=1

1. FA46C_HUMAN **Mass:** 45486 **Score:** 33 **Expect:** 10 **Matches:** 8 Protein FAM46C OS=Homo sapiens GN=FAM46C PE=2 SV=1
2. RAB7B_HUMAN **Mass:** 22725 **Score:** 32 **Expect:** 13 **Matches:** 5 Ras-related protein Rab-7b OS=Homo sapiens GN=RAB7B PE=2 SV=1
3. B3GN4_HUMAN **Mass:** 42853 **Score:** 32 **Expect:** 14 **Matches:** 7

UDP-GlcNAc:betaGal beta-1,3-N-acetylglucosaminyltransferase 4 OS=Homo sapiens GN=B3GNT4 PE=2 SV=1

1. SH3L1_HUMAN **Mass:** 12766 **Score:** 28 **Expect:** 29 **Matches:** 4

SH3 domain-binding glutamic acid-rich-like protein OS=Homo sapiens GN=SH3BGRL PE=1 SV=1

1. LIN7B_HUMAN **Mass:** 22939 **Score:** 27 **Expect:** 38 **Matches:** 5 Protein lin-7 homolog B OS=Homo sapiens GN=LIN7B PE=1 SV=1
2. SNX31_HUMAN **Mass:** 51567 **Score:** 26 **Expect:** 46 **Matches:** 9 Sorting nexin-31 OS=Homo sapiens GN=SNX31 PE=2 SV=3
3. MPP2_HUMAN **Mass:** 64882 **Score:** 26 **Expect:** 49 **Matches:** 7 MAGUK p55 subfamily member 2 OS=Homo sapiens GN=MPP2 PE=1 SV=3
4. CD014_HUMAN **Mass:** 78979 **Score:** 26 **Expect:** 50 **Matches:** 8

Uncharacterized protein C4orf14 OS=Homo sapiens GN=C4orf14 PE=1 SV=2

1. MYOF_HUMAN **Mass:** 236100 **Score:** 26 **Expect:** 52 **Matches:** 14 Myoferlin OS=Homo sapiens GN=MYOF PE=1 SV=1
2. DUPD1_HUMAN **Mass:** 25491 **Score:** 26 **Expect:** 53 **Matches:** 5

Dual specificity phosphatase DUPD1 OS=Homo sapiens GN=DUPD1 PE=2 SV=1

1. SCG2_HUMAN **Mass:** 70897 **Score:** 25 **Expect:** 60 **Matches:** 9 Secretogranin-2 OS=Homo sapiens GN=SCG2 PE=1 SV=2
2. Z876P_HUMAN **Mass:** 24053 **Score:** 25 **Expect:** 60 **Matches:** 5

Putative zinc finger protein 876 OS=Homo sapiens GN=ZNF876P PE=5 SV=3

1. SYT1_HUMAN **Mass:** 47885 **Score:** 25 **Expect:** 60 **Matches:** 7 Synaptotagmin-1 OS=Homo sapiens GN=SYT1 PE=1 SV=1
2. LNP1_HUMAN **Mass:** 21592 **Score:** 24 **Expect:** 74 **Matches:** 4 Leukemia NUP98 fusion partner 1 OS=Homo sapiens GN=LNP1 PE=2 SV=1
3. YP010_HUMAN **Mass:** 21022 **Score:** 24 **Expect:** 74 **Matches:** 4

Putative uncharacterized protein FLJ32790 OS=Homo sapiens PE=2 SV=2

1. TPST1_HUMAN **Mass:** 42560 **Score:** 24 **Expect:** 79 **Matches:** 8

Protein-tyrosine sulfotransferase 1 OS=Homo sapiens GN=TPST1 PE=2 SV=1

1. PRKRA_HUMAN **Mass:** 34839 **Score:** 24 **Expect:** 81 **Matches:** 6

Interferon-inducible double stranded RNA-dependent protein kinase activator A OS=Homo sapiens GN=PRKRA PE=1 SV=1

1. ALG3_HUMAN **Mass:** 50835 **Score:** 24 **Expect:** 83 **Matches:** 8

Dolichyl-P-Man:Man(5)GlcNAc(2)-PP-dolichyl mannosyltransferase OS=Homo sapiens GN=ALG3 PE=1 SV=1

1. HYPM_HUMAN **Mass:** 13547 **Score:** 24 **Expect:** 83 **Matches:** 4

Huntingtin-interacting protein M OS=Homo sapiens GN=CXorf27 PE=1 SV=2

1. KIF3A_HUMAN **Mass:** 80687 **Score:** 23 **Expect:** 97 **Matches:** 11 Kinesin-like protein KIF3A OS=Homo sapiens GN=KIF3A PE=1 SV=3
2. RBG1L_HUMAN **Mass:** 93366 **Score:** 23 **Expect:** 1.1e+002 **Matches:** 11

Rab GTPase-activating protein 1-like OS=Homo sapiens GN=RABGAP1L PE=1 SV=1

1. RPAP1_HUMAN **Mass:** 154198 **Score:** 23 **Expect:** 1.1e+002 **Matches:** 11

RNA polymerase II-associated protein 1 OS=Homo sapiens GN=RPAP1 PE=1 SV=2

CS025_HUMAN **Mass:** 12927 **Score:** 22 **Expect:** 1.1e+002 **Matches:** 3 UPF0449 protein C19orf25 OS=Homo sapiens GN=C19orf25 PE=1 SV=2

1. AK1A1_HUMAN **Mass:** 36892 **Score:** 22 **Expect:** 1.2e+002 **Matches:** 5 Alcohol dehydrogenase [NADP+] OS=Homo sapiens GN=AKR1A1 PE=1 SV=3
2. FAKD3_HUMAN **Mass:** 76923 **Score:** 22 **Expect:** 1.3e+002 **Matches:** 6

FAST kinase domain-containing protein 3 OS=Homo sapiens GN=FASTKD3 PE=2 SV=1

1. MCM9_HUMAN **Mass:** 128740 **Score:** 22 **Expect:** 1.3e+002 **Matches:** 13 DNA replication licensing factor MCM9 OS=Homo sapiens GN=MCM9 PE=1 SV=3
2. TR16L_HUMAN **Mass:** 40480 **Score:** 22 **Expect:** 1.3e+002 **Matches:** 6

Tripartite motif-containing protein 16-like protein OS=Homo sapiens GN=TRIM16L PE=2 SV=2

1. ATTY_HUMAN **Mass:** 51336 **Score:** 22 **Expect:** 1.3e+002 **Matches:** 6 Tyrosine aminotransferase OS=Homo sapiens GN=TAT PE=1 SV=1
2. CM035_HUMAN **Mass:** 13693 **Score:** 22 **Expect:** 1.4e+002 **Matches:** 3

Putative uncharacterized protein C13orf35 OS=Homo sapiens GN=C13orf35 PE=2 SV=1

1. COX15_HUMAN **Mass:** 46343 **Score:** 22 **Expect:** 1.4e+002 **Matches:** 6

Cytochrome c oxidase assembly protein COX15 homolog OS=Homo sapiens GN=COX15 PE=1 SV=1

1. AN32D_HUMAN **Mass:** 14911 **Score:** 22 **Expect:** 1.4e+002 **Matches:** 5

Acidic leucine-rich nuclear phosphoprotein 32 family member D OS=Homo sapiens GN=ANP32D PE=1 SV=2

1. KV307_HUMAN **Mass:** 11937 **Score:** 22 **Expect:** 1.4e+002 **Matches:** 3

Ig kappa chain V-III region GOL OS=Homo sapiens PE=1 SV=1

1. DTX3L_HUMAN **Mass:** 84585 **Score:** 22 **Expect:** 1.4e+002 **Matches:** 9 E3 ubiquitin-protein ligase DTX3L OS=Homo sapiens GN=DTX3L PE=1 SV=1
2. CA125_HUMAN **Mass:** 118637 **Score:** 22 **Expect:** 1.4e+002 **Matches:** 9 Uncharacterized protein C1orf125 OS=Homo sapiens GN=C1orf125 PE=1 SV=1
3. CP19A_HUMAN **Mass:** 58358 **Score:** 22 **Expect:** 1.4e+002 **Matches:** 7 Cytochrome P450 19A1 OS=Homo sapiens GN=CYP19A1 PE=1 SV=3
4. CU025_HUMAN **Mass:** 75999 **Score:** 21 **Expect:** 1.5e+002 **Matches:** 7 C2 domain-containing protein 2 OS=Homo sapiens GN=C2CD2 PE=1 SV=2
5. RAB36_HUMAN **Mass:** 36813 **Score:** 21 **Expect:** 1.5e+002 **Matches:** 4 Ras-related protein Rab-36 OS=Homo sapiens GN=RAB36 PE=1 SV=2
6. EHD4_HUMAN **Mass:** 61365 **Score:** 21 **Expect:** 1.6e+002 **Matches:** 6 EH domain-containing protein 4 OS=Homo sapiens GN=EHD4 PE=1 SV=1
7. CRHBP_HUMAN **Mass:** 36748 **Score:** 21 **Expect:** 1.6e+002 **Matches:** 5

Corticotropin-releasing factor-binding protein OS=Homo sapiens GN=CRHBP PE=1 SV=2

1. FOXP3_HUMAN **Mass:** 47727 **Score:** 21 **Expect:** 1.6e+002 **Matches:** 6

Forkhead box protein P3 OS=Homo sapiens GN=FOXP3 PE=1 SV=1

1. KATL1_HUMAN **Mass:** 55699 **Score:** 21 **Expect:** 1.6e+002 **Matches:** 7

Katanin p60 ATPase-containing subunit A-like 1 OS=Homo sapiens GN=KATNAL1 PE=2 SV=1

1. CA146_HUMAN **Mass:** 20633 **Score:** 21 **Expect:** 1.6e+002 **Matches:** 5 Uncharacterized protein C1orf146 OS=Homo sapiens GN=C1orf146 PE=2 SV=1
2. SNTAN_HUMAN **Mass:** 16629 **Score:** 21 **Expect:** 1.6e+002 **Matches:** 6 Sentan OS=Homo sapiens GN=SNTN PE=2 SV=1
3. GTL3B_HUMAN **Mass:** 13195 **Score:** 21 **Expect:** 1.6e+002 **Matches:** 3 Protein GTLF3B OS=Homo sapiens GN=GTLF3B PE=2 SV=2
4. ZN672_HUMAN **Mass:** 52245 **Score:** 21 **Expect:** 1.7e+002 **Matches:** 7 Zinc finger protein 672 OS=Homo sapiens GN=ZNF672 PE=2 SV=2
5. 41_HUMAN **Mass:** 97528 **Score:** 21 **Expect:** 1.8e+002 **Matches:** 8 Protein 4.1 OS=Homo sapiens GN=EPB41 PE=1 SV=4
6. SRP54_HUMAN **Mass:** 55953 **Score:** 20 **Expect:** 1.8e+002 **Matches:** 8

Signal recognition particle 54 kDa protein OS=Homo sapiens GN=SRP54 PE=1 SV=1

1. ATS10_HUMAN **Mass:** 124559 **Score:** 20 **Expect:** 1.9e+002 **Matches:** 12

A disintegrin and metalloproteinase with thrombospondin motifs 10 OS=Homo sapiens GN=ADAMTS10 PE=1 SV=2

# Search Parameters

**Type of search : Peptide Mass Fingerprint**

**Enzyme : Trypsin**

**Fixed modifications : Carbamidomethyl (C)**

**Variable modifications : Oxidation (M)**

**Mass values : Monoisotopic**

**Protein Mass : Unrestricted**

**Peptide Mass Tolerance : ± 0.5 Da**

**Peptide Charge State : 1+**

**Max Missed Cleavages : 1** **Number of queries : 52**

**Selected for scoring : 47**

**Mascot:**

http://www.matrixscience.com/
