# Supplementary material for: Identification and differential expression of serotransferrin and apolipoprotein A-I in the plasma of HIV-1 patients treated with first-line antiretroviral therapy
Source: BMC Infect Dis. 2020 Nov 27;20:898. doi: 10.1186/s12879-020-05610-6 (PMC7694411; doi:10.1186/s12879-020-05610-6)
Supplement: Supplementary file 5 — Additional file 5. Serotransferrin. [file 12879_2020_5610_MOESM5_ESM.docx]

Supplementary file-5 : Serotransferrin


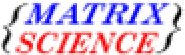
 **Mascot Search Results**

**User : sheev**

**Email : nallapeta@bdal.in**

**Search title :**

**Database : SwissProt 57.15 (515203 sequences; 181334896 residues)**

**Taxonomy : Homo sapiens (human) (20266 sequences)**

**Timestamp : 18 Feb 2020 at 08:48:58 GMT**

**Top Score : 105 for TRFE_HUMAN, Serotransferrin OS=Homo sapiens GN=TF PE=1 SV=2** **Mascot Score Histogram**

Protein score is -10*Log(P), where P is the probability that the observed match is a random event. Protein scores greater than 56 are significant (p<0.05).


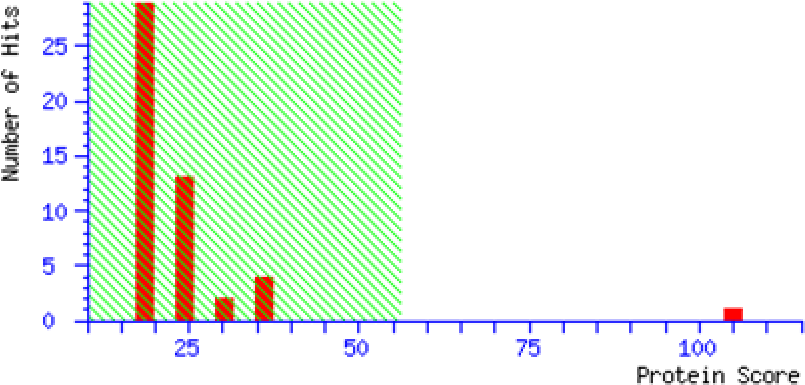


# Concise Protein Summary Report

Format As

6

Concise Protein Summary

Help

Significance threshold p<

0.05

Max. number of hits

100

Re

-

Search All

Search Unmatched

1. TRFE_HUMAN **Mass:** 79280 **Score:** **105**  **Expect:** 6.4e-007 **Matches:** 21 Serotransferrin OS=Homo sapiens GN=TF PE=1 SV=2
2. APOA1_HUMAN **Mass:** 30759 **Score:** 36 **Expect:** 5 **Matches:** 7 Apolipoprotein A-I OS=Homo sapiens GN=APOA1 PE=1 SV=1
3. CG065_HUMAN **Mass:** 17048 **Score:** 36 **Expect:** 5.2 **Matches:** 5

Uncharacterized protein C7orf65 OS=Homo sapiens GN=C7orf65 PE=2 SV=1

1. FRM4A_HUMAN **Mass:** 115958 **Score:** 34 **Expect:** 8.3 **Matches:** 13

FERM domain-containing protein 4A OS=Homo sapiens GN=FRMD4A PE=1 SV=3

1. ZN605_HUMAN **Mass:** 76666 **Score:** 33 **Expect:** 9.9 **Matches:** 9 Zinc finger protein 605 OS=Homo sapiens GN=ZNF605 PE=2 SV=1
2. IKBP1_HUMAN **Mass:** 29373 **Score:** 29 **Expect:** 23 **Matches:** 5

Interleukin-1 receptor-associated kinase 1-binding protein 1 OS=Homo sapiens GN=IRAK1BP1 PE=2 SV=1

1. KCRS_HUMAN **Mass:** 47988 **Score:** 29 **Expect:** 23 **Matches:** 6

Creatine kinase S-type, mitochondrial OS=Homo sapiens GN=CKMT2 PE=1 SV=2

1. ZN480_HUMAN **Mass:** 61022 **Score:** 26 **Expect:** 49 **Matches:** 6 Zinc finger protein 480 OS=Homo sapiens GN=ZNF480 PE=1 SV=1
2. ANKY2_HUMAN **Mass:** 50293 **Score:** 26 **Expect:** 50 **Matches:** 7

Ankyrin repeat and MYND domain-containing protein 2 OS=Homo sapiens GN=ANKMY2 PE=1 SV=1

1. CLC4K_HUMAN **Mass:** 36987 **Score:** 25 **Expect:** 58 **Matches:** 5

C-type lectin domain family 4 member K OS=Homo sapiens GN=CD207 PE=1 SV=2

1. RT22_HUMAN **Mass:** 41425 **Score:** 24 **Expect:** 81 **Matches:** 5

28S ribosomal protein S22, mitochondrial OS=Homo sapiens GN=MRPS22 PE=1 SV=1

1. DYH3_HUMAN **Mass:** 473776 **Score:** 24 **Expect:** 83 **Matches:** 27

Dynein heavy chain 3, axonemal OS=Homo sapiens GN=DNAH3 PE=2 SV=1

JOS1_HUMAN **Mass:** 23468 **Score:** 19 **Expect:** 2.7e+002 **Matches:** 3 Josephin-1 OS=Homo sapiens GN=JOSD1 PE=2 SV=1

1. TAB3_HUMAN **Mass:** 79375 **Score:** 24 **Expect:** 83 **Matches:** 9

Mitogen-activated protein kinase kinase kinase 7-interacting protein 3 OS=Homo sapiens GN=MAP3K7IP3 PE=1 SV=2

1. DD11L_HUMAN **Mass:** 102944 **Score:** 23 **Expect:** 1e+002 **Matches:** 7

Putative ATP-dependent RNA helicase DDX11-like protein OS=Homo sapiens PE=1 SV=1

1. CA087_HUMAN **Mass:** 62395 **Score:** 23 **Expect:** 1.1e+002 **Matches:** 7 Uncharacterized protein C1orf87 OS=Homo sapiens GN=C1orf87 PE=2 SV=1
2. PISD_HUMAN **Mass:** 47056 **Score:** 22 **Expect:** 1.2e+002 **Matches:** 5

Phosphatidylserine decarboxylase proenzyme OS=Homo sapiens GN=PISD PE=2 SV=3

1. DC121_HUMAN **Mass:** 51739 **Score:** 22 **Expect:** 1.3e+002 **Matches:** 5

DDB1- and CUL4-associated factor 12-like protein 1 OS=Homo sapiens GN=DCAF12L1 PE=2 SV=1

1. DDX12_HUMAN **Mass:** 107307 **Score:** 22 **Expect:** 1.3e+002 **Matches:** 8

Probable ATP-dependent RNA helicase DDX12 OS=Homo sapiens GN=DDX12 PE=2 SV=3

1. COPB_HUMAN **Mass:** 108214 **Score:** 22 **Expect:** 1.3e+002 **Matches:** 9 Coatomer subunit beta OS=Homo sapiens GN=COPB1 PE=1 SV=3
2. SCG2_HUMAN **Mass:** 70897 **Score:** 22 **Expect:** 1.4e+002 **Matches:** 10 Secretogranin-2 OS=Homo sapiens GN=SCG2 PE=1 SV=2
3. KV302_HUMAN **Mass:** 11882 **Score:** 21 **Expect:** 1.6e+002 **Matches:** 3 Ig kappa chain V-III region SIE OS=Homo sapiens PE=1 SV=1
4. SNX31_HUMAN **Mass:** 51567 **Score:** 21 **Expect:** 1.6e+002 **Matches:** 7 Sorting nexin-31 OS=Homo sapiens GN=SNX31 PE=2 SV=3
5. I17EL_HUMAN **Mass:** 38516 **Score:** 21 **Expect:** 1.6e+002 **Matches:** 4

Putative interleukin-17 receptor E-like OS=Homo sapiens GN=IL17REL PE=2 SV=2

1. SEM4C_HUMAN **Mass:** 93761 **Score:** 21 **Expect:** 1.7e+002 **Matches:** 8 Semaphorin-4C OS=Homo sapiens GN=SEMA4C PE=1 SV=2
2. LGMN_HUMAN **Mass:** 49779 **Score:** 21 **Expect:** 1.7e+002 **Matches:** 6 Legumain OS=Homo sapiens GN=LGMN PE=1 SV=1
3. YD023_HUMAN **Mass:** 16172 **Score:** 21 **Expect:** 1.8e+002 **Matches:** 4 Putative uncharacterized protein FLJ45035 OS=Homo sapiens PE=5 SV=1
4. CC014_HUMAN **Mass:** 14998 **Score:** 20 **Expect:** 1.8e+002 **Matches:** 5 Uncharacterized protein C3orf14 OS=Homo sapiens GN=C3orf14 PE=2 SV=1
5. RAB10_HUMAN **Mass:** 22755 **Score:** 20 **Expect:** 2e+002 **Matches:** 6 Ras-related protein Rab-10 OS=Homo sapiens GN=RAB10 PE=1 SV=1
6. BAD_HUMAN **Mass:** 18381 **Score:** 20 **Expect:** 2e+002 **Matches:** 3 Bcl2 antagonist of cell death OS=Homo sapiens GN=BAD PE=1 SV=3
7. RAI14_HUMAN **Mass:** 110601 **Score:** 20 **Expect:** 2e+002 **Matches:** 12 Ankycorbin OS=Homo sapiens GN=RAI14 PE=1 SV=2
8. KC1G3_HUMAN **Mass:** 51755 **Score:** 20 **Expect:** 2.2e+002 **Matches:** 6 Casein kinase I isoform gamma-3 OS=Homo sapiens GN=CSNK1G3 PE=1 SV=2
9. SSX3_HUMAN **Mass:** 21740 **Score:** 20 **Expect:** 2.2e+002 **Matches:** 3

Protein SSX3 OS=Homo sapiens GN=SSX3 PE=1 SV=2

1. ZFR_HUMAN **Mass:** 118079 **Score:** 20 **Expect:** 2.2e+002 **Matches:** 12 Zinc finger RNA-binding protein OS=Homo sapiens GN=ZFR PE=1 SV=2
2. LIPB1_HUMAN **Mass:** 114523 **Score:** 19 **Expect:** 2.3e+002 **Matches:** 10 Liprin-beta-1 OS=Homo sapiens GN=PPFIBP1 PE=1 SV=2
3. AADAT_HUMAN **Mass:** 47492 **Score:** 19 **Expect:** 2.3e+002 **Matches:** 6

Kynurenine/alpha-aminoadipate aminotransferase, mitochondrial OS=Homo sapiens GN=AADAT PE=1 SV=2

1. FHR2_HUMAN **Mass:** 31543 **Score:** 19 **Expect:** 2.4e+002 **Matches:** 3

Complement factor H-related protein 2 OS=Homo sapiens GN=CFHR2 PE=1 SV=1

1. ANGP2_HUMAN **Mass:** 57396 **Score:** 19 **Expect:** 2.5e+002 **Matches:** 5 Angiopoietin-2 OS=Homo sapiens GN=ANGPT2 PE=1 SV=1
2. TRA2A_HUMAN **Mass:** 32726 **Score:** 19 **Expect:** 2.6e+002 **Matches:** 5 Transformer-2 protein homolog alpha OS=Homo sapiens GN=TRA2A PE=1 SV=1
3. PLCB4_HUMAN **Mass:** 135518 **Score:** 19 **Expect:** 2.6e+002 **Matches:** 12

1-phosphatidylinositol-4,5-bisphosphate phosphodiesterase beta-4 OS=Homo sapiens GN=PLCB4 PE=1 SV=3

1. SPCS3_HUMAN **Mass:** 20358 **Score:** 19 **Expect:** 2.6e+002 **Matches:** 5 Signal peptidase complex subunit 3 OS=Homo sapiens GN=SPCS3 PE=1 SV=1
2. RM24_HUMAN **Mass:** 25013 **Score:** 19 **Expect:** 2.6e+002 **Matches:** 7

39S ribosomal protein L24, mitochondrial OS=Homo sapiens GN=MRPL24 PE=1 SV=1

1. EFCB1_HUMAN **Mass:** 24757 **Score:** 19 **Expect:** 2.7e+002 **Matches:** 3

EF-hand calcium-binding domain-containing protein 1 OS=Homo sapiens GN=EFCAB1 PE=2 SV=1

1. CALB1_HUMAN **Mass:** 30291 **Score:** 19 **Expect:** 2.7e+002 **Matches:** 4 Calbindin OS=Homo sapiens GN=CALB1 PE=1 SV=2
2. PXDC1_HUMAN **Mass:** 56238 **Score:** 19 **Expect:** 2.7e+002 **Matches:** 4 Plexin domain-containing protein 1 OS=Homo sapiens GN=PLXDC1 PE=1 SV=2
3. PPAC3_HUMAN **Mass:** 29771 **Score:** 19 **Expect:** 2.7e+002 **Matches:** 5

Probable lipid phosphate phosphatase PPAPDC3 OS=Homo sapiens GN=PPAPDC3 PE=2 SV=1

1. INT11_HUMAN **Mass:** 68360 **Score:** 19 **Expect:** 2.7e+002 **Matches:** 6 Integrator complex subunit 11 OS=Homo sapiens GN=CPSF3L PE=1 SV=2
2. PARVB_HUMAN **Mass:** 41745 **Score:** 19 **Expect:** 2.7e+002 **Matches:** 4 Beta-parvin OS=Homo sapiens GN=PARVB PE=1 SV=1
3. DIC_HUMAN **Mass:** 31718 **Score:** 19 **Expect:** 2.8e+002 **Matches:** 6

Mitochondrial dicarboxylate carrier OS=Homo sapiens GN=SLC25A10 PE=1 SV=2

1. CCD87_HUMAN **Mass:** 96812 **Score:** 19 **Expect:** 2.8e+002 **Matches:** 9

Coiled-coil domain-containing protein 87 OS=Homo sapiens GN=CCDC87 PE=2 SV=1

# Search Parameters

**Type of search : Peptide Mass Fingerprint**

**Enzyme : Trypsin**

**Fixed modifications : Carbamidomethyl (C)**

**Variable modifications : Oxidation (M)**

**Mass values : Monoisotopic**

**Protein Mass : Unrestricted**

**Peptide Mass Tolerance : ± 0.5 Da**

**Peptide Charge State : 1+** **Max Missed Cleavages : 1**

**Number of queries : 56**

**Selected for scoring : 30**

**Mascot:**

http://www.matrixscience.com/
