# Supplementary material for: Identification and differential expression of serotransferrin and apolipoprotein A-I in the plasma of HIV-1 patients treated with first-line antiretroviral therapy
Source: BMC Infect Dis. 2020 Nov 27;20:898. doi: 10.1186/s12879-020-05610-6 (PMC7694411; doi:10.1186/s12879-020-05610-6)
Supplement: Supplementary file 6 — Additional file 6. Protein networks and Gene ontology of serotransferrin and apolipoprotein-A1 by STRING -V 11.0. [file 12879_2020_5610_MOESM6_ESM.pdf]

Supplementary file-6

Protein networks and Gene ontology of serotransferrin and apolipoprotein-A1 by STRING - V 11.0

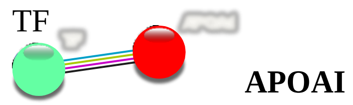

Number of nodes: 2

Number of edges: 1

Average node degree:1

Avg. local clustering coefficient: 1

Expected number of edges:0 PPI enrichment p-value: 0.0486 your network has significantly more interactions than expected (what does that mean?)

Biological Process(GO)

**GO-term**	**Description**	**Count in gene set**	**False discovery rate**
GO:0002576	Platelet degranulation	2 of 129	0.0201
GO:0031647	Regulation of protein stability	2 of 251	0.0377

Cellular Component (GO)

GO-term	Description	Count in gene set	False discovery rate
GO:0034774	Secretory granule lumen	2 of 323	0.0172
GO:0030139	Endocytic vesicle	2 of 275	0.0172
GO:0009986	Cell surface	2 of 690	0.0172
GO:0005788	Endoplasmic reticulum lumen	2 of 299	0.0172
GO:0005769	Early endosome	2 of 341	0.0172

Molecular function analysis by panther 15.0

Family ID	Mapped IDs	Family Name	PANTHER GO-Slim Molecular Function
PTHR18976:SF11	HUMAN| HGNC=600<br>UniProt KB=P02647	APOLIPOPROTEIN A-I	Cholesterol binding<br>O-acyltransferase activity<br>Enzyme activator activity<br>Phospholipid binding

Reactome pathways

Pathways	Description	Count in gene set	False discovery rate
HSA-8957275	Post-translational protein phosphorylation	2 of 106	0.0013
HSA-381426	Regulation of Insulin-like Growth Factor (IGF) transport	2 of 123	0.0013
HSA-114608	Platelet degranulation	2 of 125	0.0013
HSA-5653656	Vesicle-mediated transport	2 of 649	0.0068
HSA-382551	Transport of small molecules	2 of 706	0.0070
