# Supplementary material for: Identification and differential expression of serotransferrin and apolipoprotein A-I in the plasma of HIV-1 patients treated with first-line antiretroviral therapy
Source: BMC Infect Dis. 2020 Nov 27;20:898. doi: 10.1186/s12879-020-05610-6 (PMC7694411; doi:10.1186/s12879-020-05610-6)
Supplement: Supplementary file 7 — Additional file 7. Gene Bank accession numbers. [file 12879_2020_5610_MOESM7_ESM.docx]

Supplementary file-7 :Gene Bank accession numbers

The gene bank accession numbers were MG788713, MG788728, MG788738, MG788748 obtained from NCBI. The gene bank accession website: <https://www>.ncbi.nlm.nih.gov/ nucleotide.

LOCUS      MG788713                1289 bp    RNA    linear  VRL 21-AUG-2018

DEFINITION  HIV-1 isolate ssk154 from India pol protein (pol) gene, partial

            cds.

ACCESSION  MG788713

VERSION    MG788713

KEYWORDS    .

SOURCE      Human immunodeficiency virus 1 (HIV-1)

  ORGANISM  Human immunodeficiency virus 1

            Viruses; Ortervirales; Retroviridae; Orthoretrovirinae; Lentivirus.

REFERENCE  1  (bases 1 to 1289)

  AUTHORS  Barik,S.K., Tripathy,S.P., Mohanty,K.K., Luke,E.H.,

            Karunaianatham,R., S,M.N., Pattabiraman,S., Haribabu,H., Tandon,R.

            and Singh,T.P.

  TITLE    Drug resistant HIV-1 mutants of Agra Region, India

  JOURNAL  Unpublished

REFERENCE  2  (bases 1 to 1289)

  AUTHORS  Barik,S.K., Tripathy,S.P., Mohanty,K.K., Luke,E.H.,

            Karunaianatham,R., S,M.N., Pattabiraman,S., Haribabu,H., Tandon,R.

            and Singh,T.P.

  TITLE    Direct Submission

  JOURNAL  Submitted (16-JAN-2018) HIV/AIDS Laboratory, Division of

            Immunology, National Jalma Institute for Leprosy and Other

            Mycobacterial Diseases, Dr. M Miyazaki Marg, Agra, Uttar Pradesh

            282004, India

COMMENT    ##Assembly-Data-START##

            Sequencing Technology :: Sanger dideoxy sequencing

            ##Assembly-Data-END##

FEATURES            Location/Qualifiers

    source          1..1289

                    /organism="Human immunodeficiency virus 1"

                    /mol_type="genomic RNA"

                    /isolate="ssk154"

                    /isolation_source="human plasma from first line ART

                    failure patient"

                    /host="Homo sapiens"

                    /db_xref="taxon:11676"

                    /country="India: Agra region"

                    /collection_date="07-Oct-2016"

                    /collected_by="Sushanta and M M Alam"

                    /note="subtype: C"

    gene            <1..>1289

                    /gene="pol"

    CDS            <1..>1289

                    /gene="pol"

                    /codon_start=1

                    /product="pol protein"

                    /protein_id="AXP19112"

                    /translation="PQITLWQRPLVSIRVGGQTKEALLDTGADDTVLEEINLPGKWKP

                    KMIGGIGGFIKVRQYDXIXIEICGKXAIGTVLVGPTPINIIGRNLLTQLGCTLNFPIS

                    PIETVPVKLKPGMDGPKVKQWPLTEEKIKALTAICDEMEKEGKITKIGPENPYNTPIF

                    AIKKKDSTKWRKLVDFRELNKRTQDFWEVQLGIPHPAGLKKKKSVTVLDVGDAYFSVP

                    LYEDFRKYTAFTIPSRNNETPGIRYQYNVLPQGWKGSPAIFQASMTKILEPFRAKNPX

                    IVIYQYMDDLYVGSDLEIGQHRAKIEELREHLLKWGFTTPDKKHQKEPPFLWMGYELH

                    PDKWTVQPIQLPEKDSWTVNDIQKLVGKLNWASQIYPGIKVRQLCKLLRGTKALTDIV

                    PLTEEAELELAENREILKEPVHGVYYDPSKDLIAEIQ"

ORIGIN

        1 cctcaaatca ctctttggca rcgacccctt gtctcaataa gagtaggagg tcaaacaaaa

      61 gaggctctct tagatacagg agcagatgat actgtgttag aagaaataaa tttgccagga

      121 aaatggaaac caaaaatgat aggaggaatt ggaggtttta ttaaagtaag acaatatgat

      181 camatarcta tagaaatttg tggaaaaarg gctataggta cagtattagt agggcccaca

      241 cctatcaaca taattggaag gaatctgttg acccagcttg ggtgcacact aaattttcca

      301 atcagtccca ttgaaactgt accagtaaaa ttaaagccag gaatggatgg cccaaaggtt

      361 aaacaatggc cattaacaga agagaaaata aaagcattaa cagcaatttg tgatgaratg

      421 gaaaaagaag gaaaaattac aaaaattggg cctgaaaayc catataacac tccaatattt

      481 gccataaaaa agaaggatag yactaagtgg agaaaattag tagatttcag agaacttaat

      541 aaaagaactc aagatttttg ggaagttcaa ttaggaatac cacacccagc agggttraaa

      601 aagaaaaaat cagtgacagt actagacgtg ggggatgcat atttttcagt tcctttatat

      661 gaagacttca ggaaatatac tgcattcacc atccctagta gaaacaayga aacaccaggg

      721 attagatatc aatataatgt gctgccacag ggatggaarg gatcaccagc aatattccag

      781 gctagcatga caaaaatctt agaaccyttt agggcaaaaa acccagamat agtcatctat

      841 caatatatgg atgacttgta tgtaggatct gacttagaaa tagggcaaca tagagcaaaa

      901 atagaagagt taagagaaca tctgttaaag tggggattta ccaccccaga caagaarcat

      961 cagaaagaac ccccatttct ttggatgggg tatgaactcc atcctgacaa atggacagta

    1021 cagcctatac arttgccaga aaaggatagc tggactgtca atgatataca gaagttagtg

    1081 ggaaaattaa actgggcaag tcagatttac ccaggaatta aagtaaggca actgtgtaaa

    1141 ctccttaggg ggaccaaagc actaacagac atagtaccac taactgaaga agcagaatta

    1201 gaattggcag aaaacaggga aattctaaaa gaaccagtac atggagtata ytatgaccca

    1261 tcaaaagact tgatagctga aatacagaa

LOCUS      MG788728                1285 bp    RNA    linear  VRL 21-AUG-2018

DEFINITION  HIV-1 isolate ssk150 from India pol protein (pol) gene, partial

            cds.

ACCESSION  MG788728

VERSION    MG788728

KEYWORDS    .

SOURCE      Human immunodeficiency virus 1 (HIV-1)

  ORGANISM  Human immunodeficiency virus 1

            Viruses; Ortervirales; Retroviridae; Orthoretrovirinae; Lentivirus.

REFERENCE  1  (bases 1 to 1285)

  AUTHORS  Barik,S.K., Tripathy,S.P., Mohanty,K.K., Luke,E.H.,

            Karunaianatham,R., S,M.N., Pattabiraman,S., Haribabu,H., Tandon,R.

            and Singh,T.P.

  TITLE    Drug resistant HIV-1 mutants of Agra Region, India

  JOURNAL  Unpublished

REFERENCE  2  (bases 1 to 1285)

  AUTHORS  Barik,S.K., Tripathy,S.P., Mohanty,K.K., Luke,E.H.,

            Karunaianatham,R., S,M.N., Pattabiraman,S., Haribabu,H., Tandon,R.

            and Singh,T.P.

  TITLE    Direct Submission

  JOURNAL  Submitted (16-JAN-2018) HIV/AIDS Laboratory, Division of

            Immunology, National Jalma Institute for Leprosy and Other

            Mycobacterial Diseases, Dr. M Miyazaki Marg, Agra, Uttar Pradesh

            282004, India

COMMENT    ##Assembly-Data-START##

            Sequencing Technology :: Sanger dideoxy sequencing

            ##Assembly-Data-END##

FEATURES            Location/Qualifiers

    source          1..1285

                    /organism="Human immunodeficiency virus 1"

                    /mol_type="genomic RNA"

                    /isolate="ssk150"

                    /isolation_source="human plasma from first line ART

                    failure patient"

                    /host="Homo sapiens"

                    /db_xref="taxon:11676"

                    /country="India: Agra region"

                    /collection_date="06-Oct-2016"

                    /collected_by="Sushanta and M M Alam"

                    /note="subtype: C"

    gene            <1..>1285

                    /gene="pol"

    CDS            <1..>1285

                    /gene="pol"

                    /codon_start=1

                    /product="pol protein"

                    /protein_id="AXP19126"

                    /translation="PQITLWQRPLVTISVGGQTREALLDTGADDTVLEDINLPGKWKP

                    KMIGGIGGFIKVRQYEEVPIEICGKKAIGTVLVGPTPVNIIGRNLLTQLGCTLNFPIS

                    PIETIPVKLKPGMDGPKVKQWPLTEEKIKALTEICXEMEKEGKITKIGPENPYNTPIF

                    AIKKKDXTKWRKLVDFRELNKRTQDFWEVQLGIPHPAGLKQNKSVTVLDVGDAYFSVP

                    LDKDFRKYTAFTIPSINNZTPGIRYQYNVLPQGWKGSPAIFQSSMTRILEPFRAQNPX

                    IVIYQYVDDLYVGSDLEIEQHRAKIEELRDHLLKWGFFTPDKKXQKEPPFLWLGYELH

                    PDKWTVQPIQLPEKDSWTVNDIQKLVGKLNWASQIYPGIKVRQLCKLLRGAKALTDII

                    PLTPEAELELAENREILKEPVHGAYYDPSKDLIAEI"

ORIGIN

        1 cctcaaatca ctctttggca acgacccctt gttacaataa gcgtaggggg ccagacaaga

      61 gaggctctct tagacacagg agcagatgat acagtattag aagacataaa tttgccagga

      121 aaatggaagc caaaaatgat aggaggaatt ggaggtttta tcaaagtaag acaatatgaa

      181 gaggtaccta tagaaatttg tgggaaaaag gctataggta cagtattagt aggacccaca

      241 cctgtcaaca taattggaag aaatctgttg actcagcttg gatgcacact aaattttcca

      301 attagtccca ttgaaactat accagtaaaa ttaaagcctg gaatggatgg cccaaaggtt

      361 aaacaatggc cattgacaga agaaaaaata aaagcattaa cagaaatttg traggaaatg

      421 gaaaaggaag gaaagattac aaaaattggg cctgaaaatc catataacac tccaatattt

      481 gccataaara agaaggacrg tactaagtgg agaaaattag tagatttcag ggaacttaat

      541 aaaagaactc aagatttttg ggaagttcag ttaggaatac cacacccagc agggttaaaa

      601 cagaayaaat cagtgacagt actggatgtg ggggatgcat atttttcagt ccctctagac

      661 aaagacttta ggaaatatac tgcattcacc atacctagta taaacaatsa aacaccaggg

      721 attagatayc aatataatgt gcttccacag ggatggaagg gatcrccagc aatattccaa

      781 agtagcatga caagaatctt agagcccttt agggcacaaa atccagamat agtcatctat

      841 caatacgtgg atgacttgta tgtaggatct gacttagaaa tagagcaaca tagagcaaag

      901 atagaagaac taagagacca tctgttaaag tggggatttt tcacaccaga caagaaayat

      961 cagaaagaac ccccatttct ttggctgggg tatgaactcc atcctgacaa atggacagtr

    1021 cagcccatac agctgccaga aaargatagc tggactgtca atgatataca gaagttagtg

    1081 ggaaaattaa actgggcaag tcagatttac ccaggaatta aagtaaggca actttgtaaa

    1141 cttcttaggg gggccaaagc actaacagac ataataccac tgactccaga agcagaatta

    1201 gaattggcag agaacaggga aattctaaaa gaaccagtac atggagcata ttatgaccca

    1261 tcaaaagact tgatagctga aatac

LOCUS      MG788738                1293 bp    RNA    linear  VRL 21-AUG-2018

DEFINITION  HIV-1 isolate ssk208 from India pol protein (pol) gene, partial

            cds.

ACCESSION  MG788738

VERSION    MG788738

KEYWORDS    .

SOURCE      Human immunodeficiency virus 1 (HIV-1)

  ORGANISM  Human immunodeficiency virus 1

            Viruses; Ortervirales; Retroviridae; Orthoretrovirinae; Lentivirus.

REFERENCE  1  (bases 1 to 1293)

  AUTHORS  Barik,S.K., Tripathy,S.P., Mohanty,K.K., Luke,E.H.,

            Karunaianatham,R., S,M.N., Pattabiraman,S., Haribabu,H., Tandon,R.

            and Singh,T.P.

  TITLE    Drug resistant HIV-1 mutants of Agra Region, India

  JOURNAL  Unpublished

REFERENCE  2  (bases 1 to 1293)

  AUTHORS  Barik,S.K., Tripathy,S.P., Mohanty,K.K., Luke,E.H.,

            Karunaianatham,R., S,M.N., Pattabiraman,S., Haribabu,H., Tandon,R.

            and Singh,T.P.

  TITLE    Direct Submission

  JOURNAL  Submitted (16-JAN-2018) HIV/AIDS Laboratory, Division of

            Immunology, National Jalma Institute for Leprosy and Other

            Mycobacterial Diseases, Dr. M Miyazaki Marg, Agra, Uttar Pradesh

            282004, India

COMMENT    ##Assembly-Data-START##

            Sequencing Technology :: Sanger dideoxy sequencing

            ##Assembly-Data-END##

FEATURES            Location/Qualifiers

    source          1..1293

                    /organism="Human immunodeficiency virus 1"

                    /mol_type="genomic RNA"

                    /isolate="ssk208"

                    /isolation_source="human plasma from first line ART

                    failure patient"

                    /host="Homo sapiens"

                    /db_xref="taxon:11676"

                    /country="India: Agra region"

                    /collection_date="22-Nov-2016"

                    /collected_by="Sushanta and M M Alam"

                    /note="subtype: C"

    gene            <1..>1293

                    /gene="pol"

    CDS            <1..>1293

                    /gene="pol"

                    /codon_start=1

                    /product="pol protein"

                    /protein_id="AXP19134"

                    /translation="PQITLWQRPLVSIKIGGQTREALLDTGADDTVLEEIQLTGKWKP

                    KMIGGIGGFIKVRQYDQVPIEICGKKAIGTVLVGPTPVNIIGRNLLTHLGCTLNFPIS

                    PIETVPVKLKPGMDGPKVKQWPLTEEKIRALTEICNEMEKEGKITKIGPENPYNTPIF

                    AIERKDNTKWRKLVDFRELNKRTQDFWEVQLGIPHPAGLKKSKSMTVLDVGDAFFSVP

                    LHEDFRKYTAFTIPSMNNETPGIRYQYNVLPQGWKGSPAXFQTSMTKILEPFRAQNPE

                    LVIYQYVDDLYVGSDLEIGKHRAKIEELRNHLLKWGFTTPDKKHQKEPPFLWMGYELH

                    PDKWTVQPIQLPEKDSWTVNDIQKLVGKLNWASQIYPGIKVRQLCRLLRGTKALTDIV

                    TLTEEAELELAENREILKEPVHGVYYDPSKDLIAEIQKQ"

ORIGIN

        1 cctcagatca ctctttggca gcgacccctt gtctcaataa aaataggggg ccagacaaga

      61 gaggctctct tagacacagg agcagatgat acagtattag aagaaataca gttgacggga

      121 aaatggaaac caaaaatgat aggaggaatt ggaggtttta tcaaagtaag acaatatgat

      181 caagtaccta tagaaatttg tggaaaaaag gctataggta cagtattagt gggacccaca

      241 cctgtcaaca taattggaag aaatctgttg actcaccttg gatgcacact aaattttcca

      301 attagtccca ttgaaactgt accagtaaaa ttaaaaccag gaatggatgg cccaaaagtt

      361 aaacaatggc cattgacaga agaaaaaata agagcattaa cagaaatttg taatgaaatg

      421 gaaaaggaag gaaaaattac aaaaattggg cctgaaaatc catataacac tcctatattt

      481 gctatagaaa gaaaggacaa tactaaatgg agaaaattag trgatttcag ggaacttaat

      541 aaaagaactc aagacttttg ggaagtccaa ttaggaatac cacacccagc agggttaaaa

      601 aagagcaaat caatgacagt actagatgtg ggggatgcat ttttttcagt tcctttacat

      661 gaggacttca ggaagtatac tgcattcacc atacctagta tgaacaatga aacaccaggg

      721 attagatatc aatataacgt gcttccacag ggatggaaag gatcaccagc artatttcag

      781 actagcatga caaaaatctt agaacccttt agggcacaaa atccagaatt agtcatctat

      841 caatatgtgg atgacttata tgtaggatct gacctagaaa tagggaaaca tagagcaaag

      901 atagaggagt taagaaacca tctgttaaag tgggggttta ccacaccaga caagaaacat

      961 cagaaagaac ctccatttct ttggatgggg tatgaactcc atcctgacaa atggacagta

    1021 cagcctatac agttgccaga aaaggatagc tggactgtca atgatataca aaagttagtg

    1081 ggaaaattaa actgggcgag tcaaatttac ccagggatta aagtgagaca actttgtaga

    1141 ctccttaggg gaaccaaagc actaacagac atagtaacat taactgaaga agcagaatta

    1201 gaattggcag aaaacaggga aattctaaaa gaaccagtac atggagtata ttatgaccca

    1261 tcaaaagact tgatagctga aatacaaaaa cag

LOCUS      MG788748                1270 bp    RNA    linear  VRL 21-AUG-2018

DEFINITION  HIV-1 isolate ssk32 from India pol protein (pol) gene, partial cds.

ACCESSION  MG788748

VERSION    MG788748

KEYWORDS    .

SOURCE      Human immunodeficiency virus 1 (HIV-1)

  ORGANISM  Human immunodeficiency virus 1

            Viruses; Ortervirales; Retroviridae; Orthoretrovirinae; Lentivirus.

REFERENCE  1  (bases 1 to 1270)

  AUTHORS  Barik,S.K., Tripathy,S.P., Mohanty,K.K., Luke,E.H.,

            Karunaianatham,R., S,M.N., Pattabiraman,S., Haribabu,H., Tandon,R.

            and Singh,T.P.

  TITLE    Drug resistant HIV-1 mutants of Agra Region, India

  JOURNAL  Unpublished

REFERENCE  2  (bases 1 to 1270)

  AUTHORS  Barik,S.K., Tripathy,S.P., Mohanty,K.K., Luke,E.H.,

            Karunaianatham,R., S,M.N., Pattabiraman,S., Haribabu,H., Tandon,R.

            and Singh,T.P.

  TITLE    Direct Submission

  JOURNAL  Submitted (16-JAN-2018) HIV/AIDS Laboratory, Division of

            Immunology, National Jalma Institute for Leprosy and Other

            Mycobacterial Diseases, Dr. M Miyazaki Marg, Agra, Uttar Pradesh

            282004, India

COMMENT    ##Assembly-Data-START##

            Sequencing Technology :: Sanger dideoxy sequencing

            ##Assembly-Data-END##

FEATURES            Location/Qualifiers

    source          1..1270

                    /organism="Human immunodeficiency virus 1"

                    /mol_type="genomic RNA"

                    /isolate="ssk32"

                    /isolation_source="human plasma from first line ART

                    failure patient"

                    /host="Homo sapiens"

                    /db_xref="taxon:11676"

                    /country="India: Agra region"

                    /collection_date="09-Sep-2016"

                    /collected_by="Sushanta and M M Alam"

                    /note="subtype: C"

    gene            <1..>1270

                    /gene="pol"

    CDS            <1..>1270

                    /gene="pol"

                    /codon_start=1

                    /product="pol protein"

                    /protein_id="AXP19143"

                    /translation="PQITLWQRPLVAIKVGGQTKEALLDTGADDTVLEEXNLPGKWKP

                    KMIGGIGGFIKVRQYDQIPIEICGKKAIGTVLVGPTPINIIGRNMLTQLGCTLNFPIS

                    PIETVPVKLKPGMDGPXVKQWPLTEEKIXALTEICNELEKEGKIXKIGPENPYNTPIF

                    AIKKKDSTKWRKIVDFRELNKRTQDFWEIQLGIPHPXGLRKXKSVTILDVGDAYFSXP

                    LDEDFRKYTAFTIPSTNNETPGIRYQYNVLPQGWKGSPAIFQSSMTXILEPFRAKNPE

                    IVICQYVDDLYVASDLEIGQHRAKIEELRNHLLKWGFXTPDKKYQKEPPFLWMGYELH

                    PDKWTVQPIQLPEKDSWTVNDIQKLVGKLNWASQIYPGIKVRQLCKLJRGTKALTDIV

                    PLTAEAELELAENREILREPVHGVYYDPSKD"

ORIGIN

        1 cctcaaatca ctctttggca gcgacccctt gtcgcaataa aagtaggggg tcagacaaag

      61 gaggctctct tagacacagg agcagatgat acagtattag aagaaatraa tttgccagga

      121 aaatggaaac caaaaatgat aggaggaatt ggaggcttta tcaaagtaag acaatatgat

      181 caaataccta tagaaatttg tggaaaaaag gctataggta cagtattagt gggacccaca

      241 cctatcaaca taattggrag aaatatgttg actcagcttg gatgcacact aaattttcca

      301 atcagtccca ttgaaactgt accagtaaaa ttaaagccag gratggaygg cccaarrgtt

      361 aaacaatggc cattgacaga agagaaaata aragcattaa cagaaatttg taatgaattg

      421 gagaaggaag gaaaaatcwc aaaaattggg cctgaaaatc catataacac tccaatattt

      481 gccataaaaa agaaggacag tactaagtgg agaaaaatag tagacttcag ggaactcaat

      541 aaaaggactc aagatttttg ggaaattcaa ttaggaatac cacacccags agggttaaga

      601 aagmataagt cagtgacaat actagatgtg ggggatgctt atttttcart tcctttagat

      661 gaagacttca ggaagtatac tgcattcacc atacctagta caaacaatga aacaccaggr

      721 attaggtatc aatataatgt gcttccacaa ggatggaaag gatcaccagc aatattccag

      781 agtagcatga caaraatctt agagcccttt agagcaaaaa acccagaaat agtcatctgt

      841 caatatgtgg atgacttata tgtagcatct gacttagaaa tagggcaaca tagagcaaaa

      901 atagaagagt taagaaacca tctgttaaaa tgggggtttt wcacaccaga caagaaatat

      961 cagaaagaac ctccatttct ttggatgggg tatgaactcc atccggacaa atggacagta

    1021 cagcctatac agctgccaga aaaggatagc tggactgtca atgatataca gaagttagtg

    1081 ggaaaattaa actgggcaag tcagatttac ccaggaatca aagtraggca actttgtaaa

    1141 ctcmttagrg ggaccaaagc actaacagac atagtaccac taactgcaga agcagaatta

    1201 gaattggcag aaaacaggga aattctaaga gaaccwgtac atggggtata ttatgaccca

    1261 tcaaaagact
