# Supplementary material for: Identification and differential expression of serotransferrin and apolipoprotein A-I in the plasma of HIV-1 patients treated with first-line antiretroviral therapy
Source: BMC Infect Dis. 2020 Nov 27;20:898. doi: 10.1186/s12879-020-05610-6 (PMC7694411; doi:10.1186/s12879-020-05610-6)
Supplement: Supplementary file 8 — Additional file 8: Figure S1. Original image of 10% SDS-PAGE of plasma proteins extracted from HIV-1 infected human and their purification by Aurum serum mini kit (Bio-Rad, USA). Lane 1: Proteins marker; Lane 2, 3, 4: Before purification; Lane 5, 6: After purification. Fig. 2 (a) and (b): Original 2D gel electrophoresis of purified proteins from human plasma samples of HIV-1. [file 12879_2020_5610_MOESM8_ESM.docx]

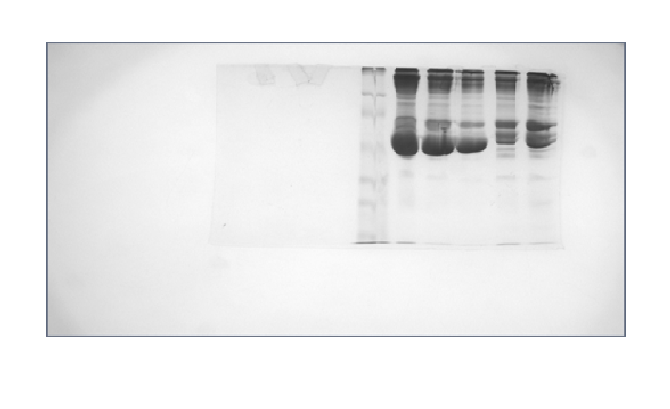


Fig. 1: Original image of 10% SDS-PAGE of plasma proteins extracted from HIV-1 infected human and their purification by Aurum serum mini kit (Bio-Rad, USA). Lane 1: Proteins marker; Lane 2, 3, 4: Before purification; Lane 5, 6: After purification

Figure-2 (a) and (b) :Original 2D gel electrophoresis of purified proteins from human plasma samples of HIV-1


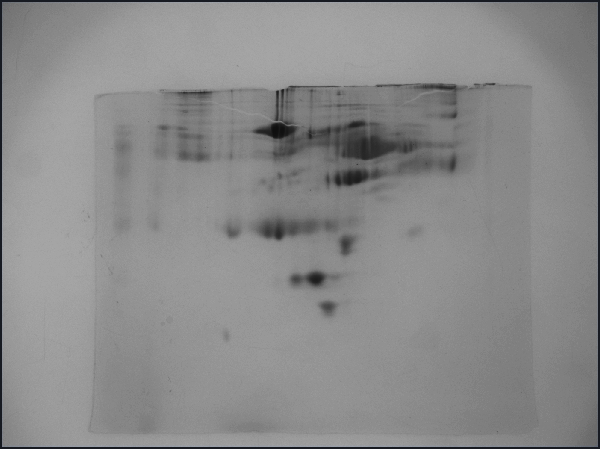


(a)Treatment responder(control)


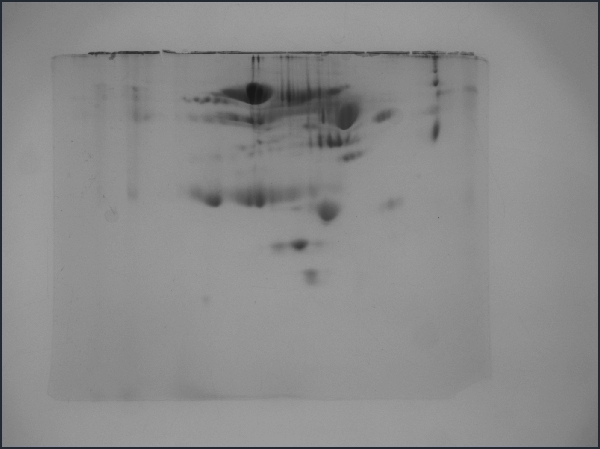


(b) Treatment failure( test)
